# UGT1A1*6 mutation associated with the occurrence and severity in infants with prolonged jaundice

**DOI:** 10.3389/fped.2022.1080212

**Published:** 2022-12-20

**Authors:** Zhe Yang, Fen Lin, Jia-Xin Xu, Hui Yang, Yong-Hao Wu, Zi-Kai Chen, He Xie, Bin Huang, Wei-Hao Lin, Jian-Peng Wu, Yu-Bin Ma, Jian-Dong Li, Li-Ye Yang

**Affiliations:** ^1^ Department of Pediatrics, Chaozhou Central Hospital Affiliated to Southern Medical University, Chaozhou, China; ^2^ Precision Medical Center, Chaozhou Central Hospital Affiliated to Southern Medical University, Chaozhou, China; ^3^ Department of Laboratory Medicine, School of Medicine, Yangtze University, Jingzhou, China; ^4^ School of Food Engineering and Biotechnology, Hanshan Normal University, Chaozhou, China; ^5^ Precision Medical Lab Center, People’s Hospital of Yangjiang, Yangjiang, China

**Keywords:** *UGT1A1*, prolonged jaundice, G6PD deficiency, breast feeding, neonatal hyperbilirubinemia

## Abstract

**Background:**

This study aimed to investigate the influence of a variant of the *UGT1A1* gene on the occurrence and severity of prolonged jaundice in Chinese infants at term.

**Methods:**

175 infants with prolonged jaundice and 149 controls were used in this retrospective case-control study. The infants with prolonged jaundice were subdivided into the mild-medium and severe jaundice groups (TSB ≥ 342 µmol/L). The frequency and genotype distribution of the *UGT1A1* and *G6PD* genes, and clinical parameters including sex, birth weight, delivery mode, gestational age, and feeding mode, were analyzed, and the differences in the parameters between the two groups were compared.

**Results:**

The allele frequency of UGT1A1*6 in the prolonged jaundice group was higher than that in the control group. Similarly, it was also higher in the severe jaundice group than in the mild-medium jaundice group. Homozygous and heterozygous UGT1A1*6 were also found more frequently in the prolonged jaundice group than in the control group. Exclusive breastfeeding, homozygous and heterozygous forms of UGT1A1*6 were significant risk indicators for prolonged jaundice. Moreover, UGT1A1*6 was the best predictor of prolonged severe jaundice.

**Conclusion:**

UGT1A1*6 appears to be a risk factor for prolonged jaundice with hyperbilirubinemia in term infants of Chinese ancestry who are exclusively breastfed.

## Background

The UDP-glucuronosyltransferase 1A1 (*UGT1A1*) gene encodes UDP-glucuronosyltransferase (UDPGT), an enzyme that plays a crucial role in the metabolism of bilirubin. The *UGT1A1* variants in hereditary unconjugated hyperbilirubinemia are distributed differently in different ethnicities and regions (1, 2). The homozygous mutation of the TATA box (A(TA)_6_TAA > A(TA)_7_TAA, UGT1A1*28, rs8175347) is identified as the primary genetic basis of Gilbert's syndrome in Caucasians and Africans (1). The incidence of c.211G > A (UGT1A1*6) in East Asia including China, Japan, and Korea, is high (16%–21%) (1, 2). The role of c.211G > A in prolonged jaundice was found in Japan and Iran (3, 4). Studies on the effects of c.211G > A on infants suffering from prolonged jaundice, especially severe prolonged jaundice, are limited in China.

Clinically, prolonged unconjugated jaundice was associated with glucose-6-phosphate dehydrogenase (G6PD) deficiency, breastfeeding, extravasated blood, sepsis, urinary tract infection, and hypothyroidism (5). The present study aimed to explore the contribution of the UGT1A1*6 variant to prolonged jaundice in term infants of Chinese ancestry. Meanwhile, we evaluated the potential confounding factors including sex, birth weight, delivery mode, gestational age, and the method of feeding. Our findings might provide information for assessing the risk of prolonged jaundice with hyperbilirubinemia, and aid genetic counseling in term infants of Chinese ancestry.

## Materials and methods

### Clinical data and specimen collection

This retrospective case-control study was carried out at Chaozhou Central Hospital affiliated to Southern Medical University, China. In-hospital neonates from Department of Neonatology or General Pediatrics from February 2012 to November 2019 were selected; 175 cases (110 males and 65 females) were in the prolonged jaundice group, while 149 cases (78 males and 71 females) were set as the control group. All infants were full-term, had gestational age greater than 37 weeks, and weighed more than 2,500 g.

The diagnostic criteria for neonatal unconjugated hyperbilirubinemia were formulated based on previous studies (6, 7). Prolonged jaundice was defined as the presence of unconjugated hyperbilirubinemia beyond 14 days with total serum bilirubin (TSB) levels >150 µmol/L, or >100 µmol/L beyond 28 days of age (6). Severe hyperbilirubinemia was defined as prolonged jaundice newborns with TSB ≥ 342 µmol/L. The inclusion criteria for the control group were infants more than 14 days old without pathological jaundice, and hospitalized for mild pneumonia, umbilical infection, or other non-jaundice causes. The exclusion criteria were neonates with hemolysis due to incompatibility of the ABO or Rh blood group, sepsis, thalassemia, extravascular hemolysis, serious maternal diseases, organ dysfunction, and congenital malformation with clear etiology, or liver disease that might affect the total serum bilirubin level. These conditions were evaluated based on the medical history of the patients, clinical and laboratory tests such as whole blood cell count, liver function tests, reticulocyte count, blood groups and subgroups, and Coombs test.

All infants received standard clinical care. After conducting a routine clinical test, the remaining ethylenediaminetetraacetic acid (EDTA) anticoagulant blood was obtained. We stored the remaining blood from the neonates in the biobank of our hospital at −40°C for further analysis. Information on sex, age, birth weight, gestational age, delivery mode, feeding pattern, and bilirubin level was recorded at admission before phototherapy. The phototherapy protocol, reviewed from medical records, was based on the American Academy of Pediatrics guidelines (2004) (7).

The Medical Ethics Committee of Chaozhou Central Hospital approved this study (Approval No. 2011021 and No. 2015001). The samples of this retrospective case-control investigation were collected after standardized diagnosis and treatment; Written informed consent to participate in this study was provided by the participants' legal guardian/next of kin.

### *G6PD* gene analysis

The G6PD-deficient samples, determined by the clinically approved G6PD enzyme quantification assay (Guangzhou Micky Medical Instrument Co., Guangdong, China), were used for molecular analysis (8). Extraction of the genomic DNA and detection of the common *G6PD* mutation, including C.95A > G (Gaohe), C.871G > A (Viangchan), C.1004C > T (Foshan), C.1024C > T (Chinese-5), C.1376G > T (Canton), and C.1388G > A (Kaiping), were performed using a kit (Yaneng Biotechnology Limited Corp., Shenzhen, China) (8). For samples highly suspected of having G6PD gene mutations but not detected with this kit, 10 pairs of primers were designed for amplifying and sequencing exons 2–13, and the sequences were compared to those in NCBI BLAST (http://blast.ncbi.nlm.nih.gov/Blast.cgi) (8).

### Analysis of the *UGT1A1* polymorphism

Genomic DNA from peripheral blood samples was extracted using the blood genomic DNA extraction kits (Yaneng Biotechnology Limited Corp., Shenzhen, China). The primers were designed for exon 1 and the flanking sequence of the *UGT1A1* gene; PCR amplification was performed using the TaKaRa Taq enzyme. The reaction conditions were 95°C for 5 min, followed by 94°C for 30 s, 60°C for 40 s, 72°C for 1 min, 35 cycles, and 72°C extension for 10 min. Finally, the sequences of the samples were analyzed using an ABI Genetic Analyzer (9).

### Statistical analysis

Bilirubin levels at admission, gestational age, and birth weight were compared between two groups by conducting *T*-tests. To assess factors such as the ratio of male to female, the delivery method, the feeding methods, and the variation frequency of the *G6PD* and *UGT1A1* genes, the *χ*^2^ test or Mann–Whitney *U* test was performed. The odds and odds ratios of prolonged jaundice and severe hyperbilirubinemia (TSB ≥ 342 µmol/L), associated with *UGT1A1*, *G6PD*, sex, weight at birth, delivery mode, gestational age, and feeding methods, were analyzed with multiple logistic regression performance. SPSS16.0 was used to conduct all statistical analyses, and all differences between groups were considered to be statistically significant at *P *< 0.05.

## Results

### Demographic and clinical characteristics

**Figure 1:**
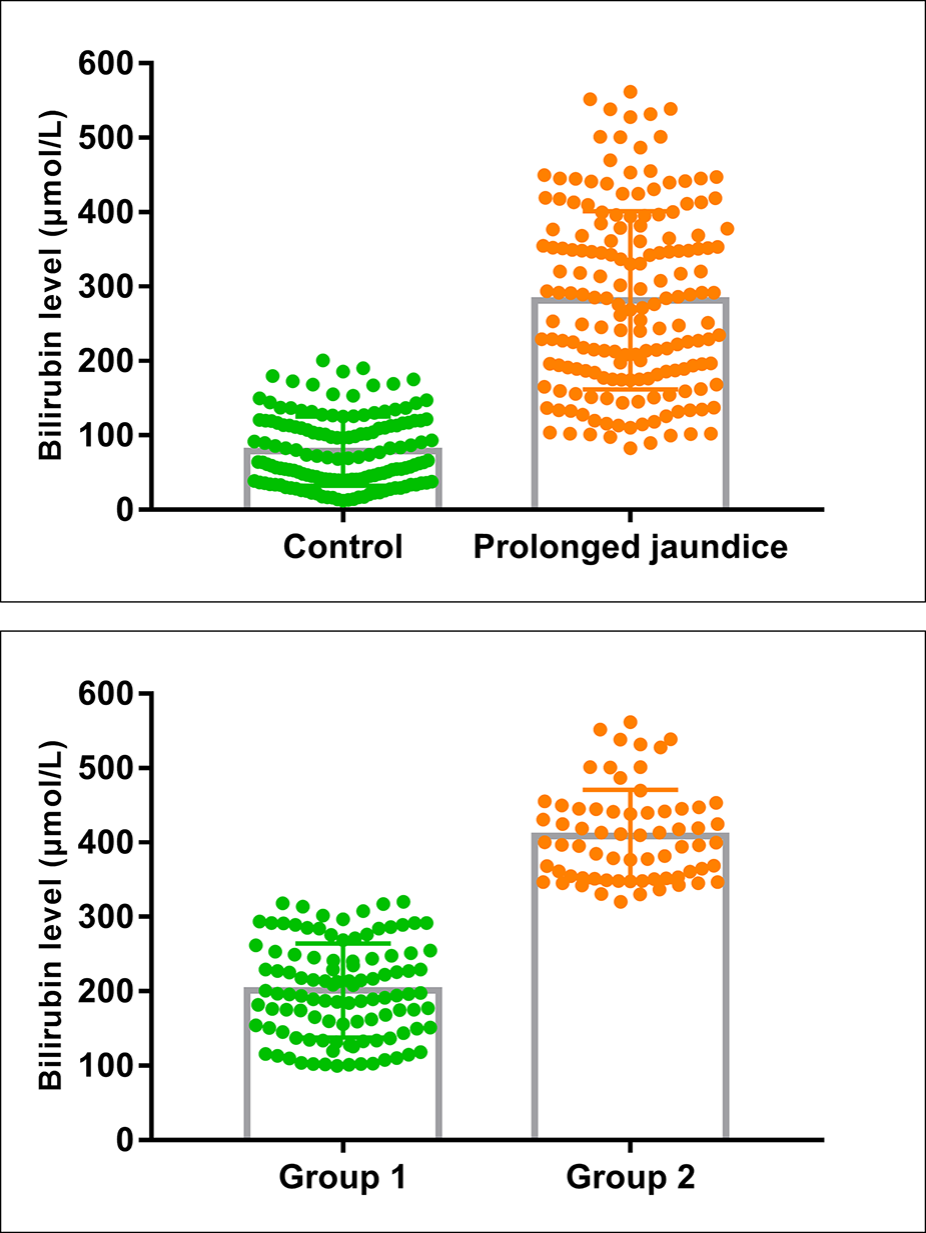
Bilirubin levels in the case and control groups. (Control, *n* = 145; Prolonged jaundice, *n* = 175; Group 1: mild-medium jaundice, *n* = 107; group 2: severe jaundice, *n* = 68).

In total, data from 324 infants were included in the study, 175 cases (110 males and 65 females) were classified in the prolonged jaundice group and 149 cases (78 males and 71 females) were in the control group. The clinical characteristics of the infants are presented in Table 1. The sex ratio, gestational age, birth weight, and delivery mode ratio between the two groups were not significantly different (*P *> 0.05); however, feeding methods differed significantly (*P *< 0.001). In addition, the bilirubin levels of the infants in the jaundice group were higher than those of the infants in the control group (Table 1 and Figure 1).

**Table 1:** Clinical characteristics and analysis of influencing factors for prolonged jaundice.

Clinical characteristics	Prolonged jaundice (*n* = 175)	Control (*n* = 149)	*P-*value	Adjusted OR (95% CI)	*P-*value
Sex					
Male	110 (62.9)	78 (52.3)	0.056	reference	NA
Female	65 (37.1)	71 (47.7)		0.57 (0.34–0.98)	0.560
Gestational age (weeks)	38.72 ± 1.37	39.00 ± 1.55	0.102	0.87 (0.74–1.03)	0.105
Birth weight (kg)	3.14 ± 0.49	3.23 ± 0.38	0.090	1.01 (0.10–1.02)	0.197
Delivery mode					
Vaginal	115 (65.7)	84 (56.4)	0.085	reference	NA
Cesarean	60 (34.3)	65 (43.6)		0.77 (0.45–1.32)	0.337
Feeding					
Formula feeding	41 (23.4)	77 (51.7)	<0.001	reference	NA
Breast feeding	84 (48.0)	31 (20.8)		2.27 (1.19–4.34)	0.012
Mix feeding	50 (28.6)	41 (27.5)		0.38 (0.20–0.73)	0.060
Maximum TSB (µmol/L)	275.5 (184.0–368.8)	68.1 (39.7–114.9)	<0.001	NA	NA
G6PD deficiency	6 (3.4)	0 (0)	0.033	2.48 (0)	0.999
UGT1A1*6 (c.211G > A)					
G/G	72 (41.1)	117 (78.5)	<0.001	reference	NA
G/A	82 (46.9)	31 (20.8)		4.43 (2.53–7.74)	0.000
A/A	21 (12.0)	1 (0.7)		63.10 (5.30–751.63)	0.001

Data are presented as *n* (%), mean ± standard deviation, or median (95% Confidence Interval). NA, not applicable; OR, odds ratio.

The infants with prolonged jaundice (*n* = 175) were divided into mild-medium jaundice group 1 (TSB < 342 µmol/L) and severe jaundice group 2 (TSB ≥ 342 µmol/L) (Figure 1). The clinical characteristics, including sex, age, gestational age, birth weight, delivery mode, and feeding method, did not differ significantly between these two groups (*P *> 0.05) (Table 2). Some infants (52/107, 48.5%) in group 1 and all infants in group 2 (68/68, 100%) received phototherapy (*χ*^2^ = 50.974, *P *< 0.001). Three infants in group 2 developed bilirubin encephalopathy, with the highest total bilirubin levels of 469.9 µmol/L, 527.9 µmol/L, and 561.9 µmol/L, respectively.

**Table 2:** Clinical characteristics and analysis of influencing factors for severe prolonged jaundice (TSB ≥ 342 µmol/L).

Clinical characteristics	Group1 (*n* = 107) (TSB < 342 µmol/L)	Group2 (*n* = 68) (TSB ≥ 342 µmol/L)	*P-*value	Adjusted OR (95% CI)	*P-*value
Sex					
Male	71 (66.4)	40 (58.8)	0.313	reference	NA
Female	36 (33.6)	28 (41.2)		1.67 (0.99–2.82)	0.056
Age (days)	20.96 ± 7.70	18.78 ± 7.70	0.065	0.97 (0.35–2.74)	0.956
Birth weight (kg)	3.11 ± 0.35	3.24 ± 0.50	0.058	0.96 (0.83–1.11)	0.535
Gestational age (weeks)	38.75 ± 1.50	38.69 ± 1.17	0.786	1.00 (0.98–1.01)	0.782
Delivery mode					
Vaginal	73 (68.2)	42 (61.8)	0.380	reference	NA
Cesarean	34 (31.8)	26 (38.2)		1.29 (0.64–2.62)	0.474
Phototherapy	52 (48.5)	68 (100.0)	<0.001	NA	NA
Feeding					
Formula feeding	26 (24.3)	15 (22.1)	0.940	reference	NA
Breast feeding	51 (47.7)	33 (48.5)		0.67 (0.28–1.60)	0.366
Mix feeding	30 (28.0)	20 (29.4)		0.86 (0.39–1.92)	0.714
MaximumTSB (µmol/L)	196.0 (149.9–249.4)	398.3 (352.0–445.2)	<0.001	NA	NA
G6PD deficiency	3 (2.8)	3 (4.4)	0.679	1.35 (0.22–0.85)	0.748
UGT1A1*6 (c.211G > A)					
** **G/G	58 (54.2)	14 (20.6)	<0.001	reference	NA
** **G/A	42 (39.3)	40 (58.8)		4.01 (1.91–8.45)	<0.001
** **A/A	7 (6.5)	14 (20.6)		8.80 (2.84–27.23)	<0.001

Data are presented as *n* (%), mean ± standard deviation, or median (95% Confidence Interval).

NA, not applicable; OR, odds ratio.

### Mutation analysis of *UGT1A1* and *G6PD*

The genotype frequencies of homozygous and heterozygous c.211G > A variant of UGT1A1*6 were 12.0% (21/175) and 46.9% (82/175) in the case group, respectively, which were higher than those of the control group (Table 1). The allele frequency of the c.211G > A variant of UGT1A1*6 in the prolonged jaundice group was 58.9% while it was 21.5% in the control group (*χ*^2 ^ = 50.15, *P* < 0.001). Furthermore, the allele frequencies of c.211G > A in groups 1 and 2 were 46% and 79%, respectively (*χ*^2^ = 21.66, *P *< 0.001) (Table 2). The prevalence of homozygous and heterozygous UGT1A1*6 in group 1 was 6.5% (7/107) and 39.3% (42/107), respectively; and in group 2, their prevalence was 20.6% (14/68) and 58.8% (40/68), respectively (Table 2).

G6PD deficiency was identified in 6 cases of prolonged jaundice group (175 cases) by enzyme assay and no G6PD deficiency case was identified in the control group. The G6PD genotypes in the prolonged jaundice group were c.1388 G > A (2/6), c.1376 G > T (2/6), c.517 T > C (NanKang) (1/6), and c.95 A > G (1/6), respectively. The frequency of G6PD deficiency differed significantly between the prolonged jaundice group (3.4%, 6/175) and the control group (0%, 0/149) (*χ*^2^ = 5.21, *P *= 0.033) (Table 1).

Of the 68 infants with severe prolonged jaundice, three suffered from bilirubin encephalopathy, with peak bilirubin values of 469–561 µmol/L, two cases were homozygous 211G > A with *G6PD* mutation (c.1376 G > T and c.1388 G > A, respectively), and one was heterozygous 211G > A with G6PD deficiency (c.95 A > G).

### Evaluation of risk factors for multiple logistic regression

Infants with prolonged jaundice, who were only breastfed, had a higher risk than formula-fed infants (OR = 2.27, 95% CI: 1.19–4.34, *P *= 0.012). Additionally, both homozygous (OR = 63.10, 95% CI: 5.30–751.63, *P *= 0.001) and heterozygous forms (OR = 4.43, 95% CI: 2.53–7.74, *P *= 0.000) of c.211G > A were significant risk assessment indicators for prolonged jaundice (Table 1). As summarized in Table 2, homozygous (OR = 8.80, 95% CI, 2.84–27.23, *P *< 0.001) and heterozygous (OR = 4.01.95% CI, 1.91–8.45, *P *< 0.001) UGT1A1*6 (c.211G > A) was the most significant predictor of severe prolonged jaundice (TSB ≥ 342 µmol/L).

## Discussion

Our results showed that the allele frequency of UGT1A1*6 was significantly different between the prolonged jaundice group (58.9%; 103/175) and the control group (21.5%; 32/149). Additionally, we found that heterozygous and homozygous forms of the UGT1A1*6 aggravated bilirubin levels in infants with prolonged jaundice. The variant UGT1A1*6 contributed to prolonged jaundice, consistent with the findings of previous studies (4, 6). A similar study in Taipei conducted *by Weng* et al*.* showed that UGT1A1*6 played a crucial role in prolonged jaundice (10). However, they only studied 30-day healthy infants, including preterm neonates, and severe prolonged jaundice was not included (10). *Maruo* et al*.* showed that UGT1A1*6, especially the homozygous variant, was associated with prolonged unconjugated hyperbilirubinemia, similar to our results (11). The variation 211 G >** **A leads to the transition of amino acid 71 from glycine to arginine, and the homozygous and heterozygous variants regulate the UDPGT activity by decreasing approximately 32.2%–60.2% of the enzyme activity, delaying bilirubin regression in infants with jaundice (6).

In this study, we also assessed multiple risk factors for prolonged jaundice in infants of Chinese descent, especially for severe jaundice, and we found that exclusive breastfeeding played a more significant role than other clinical parameters (sex, birth weight, gestational age, and delivery mode) for TSB levels. However, breastfeeding might not exacerbate the severity of prolonged jaundice as a risk factor. 5α-pregnane-3α and 20β-diol in breast milk could inhibit UGT1A1*6 bilirubin glucuronidation activity (11). Breast milk contains lipoprotein lipase, which produces nonesterified free fatty acids, thus decrease conjugation and excretion of bilirubin (12). Increased colostral interleukin-1β (IL-1β) concentrations in breast milk may repress genes involved in bilirubin metabolism (13). Thus, prolonged unconjugated hyperbilirubinemia may develop in infants with UGT1A1*6 who are fed with breast milk (10, 11, 14). While some substances in breast milk may be responsible for jaundice, on the other hand, there is an irrefutable evidence-based advantages conferred by continued breastfeeding, the breastfed infant benefits from fewer infections, increased brain growth and cognition, as well as the prospect of genetic modification of certain diseases (15). Hence, breastfeeding is encouraged even though breastfed infants experience more jaundice than those not breastfed (15).

Preterm, breastfeeding, and UGT1A1*6 are the primary risk factors for prolonged jaundice (10)*.* For preterm neonates with prolonged jaundice, immature bilirubin metabolism has a stronger effect than genetic factors (4, 10). In this study, we investigated full-term infants with more mature bilirubin metabolism, and feeding patterns and genetic factors were significant predictors for prolonged jaundice. Some studies showed that *UGT1A1* was more important in females than males for developing prolonged hyperbilirubinemia due to the higher expression of *UGT1A1* in the female liver (6). In our study, however, sex was not a significant indicator of prolonged jaundice.

This was the first study to perform a detailed analysis of the influencing factors of severe prolonged jaundice (TSB ≥ 342 µmol/L) in mainland China. In severe prolonged jaundice, genetic regulation plays a key role in bilirubin metabolism, and homozygous and heterozygous UGT1A1*6 can aggravate the TSB levels of prolonged jaundice, the homozygous variant is more dominant than the heterozygous variant. UGT1A1*6 was also the most significant predictor of severe prolonged jaundice (TSB ≥ 342 µmol/L). Studies on UGT1A1*6 polymorphism and the severity of prolonged jaundice are limited. A study found that UGT1A1*6 homozygotes contributed to prolonged jaundice of breastfed East Asian infants, and serum bilirubin in homozygous neonates was considerably higher than that in heterozygous neonates (11). Our previous study on individuals from southern China found that the homozygous or heterozygous UGT1A1*6 was closely associated with the severity of bilirubin in the Chinese neonatal cohort (16). These studies suggest that UGT1A1*6 is a significant risk factor for severe prolonged jaundice in infants from southern China.

Most homozygous carriers of UGT1A1*6 have benign presentation without severe impairment of liver function, and have a good prognosis after follow-up (2). Therefore, we speculated that these cases with severe prolonged hyperbilirubinemia might have Gilbert syndrome in infancy. Further follow-up is required to determine whether recurrent jaundice occurs in adolescence (11).

This article emphasizes the importance of UGT1A1*6 in prolonged jaundice, especially for severe hyperbilirubinemia in Chinese term infants. We found that UGT1A1*6 was an important genetic modifier for prolonged jaundice in infants with severe hyperbilirubinemia (TSB ≥ 342 µmol/L) in mainland China. Our study improved the understanding of prolonged jaundice in the pediatric field, and provided valuable information for the etiological analysis and genetic counseling for prolonged hyperbilirubinemia.

## Data Availability

The original datasheet used in the study are deposited and can be accessed publicly at https://GitHub.com, JingZhunLab/-neonatal-jaundice, further inquiries can be directed to the corresponding author/s.
